# Ultrasound Improves Gallbladder Contraction Function: A Non-Invasive Experimental Validation Using Small Animals

**DOI:** 10.3390/bioengineering12070716

**Published:** 2025-06-30

**Authors:** Run Guo, Tian Chen, Fan Ding, Li-Ping Liu, Fang Chen, Gang Zhao, Bo Zhang

**Affiliations:** 1 Department of Ultrasound in Medicine, Shanghai East Hospital, School of Medicine, Tongji University, Shanghai 200120, China; 2033243@tongji.edu.cn (R.G.); chentian109@163.com (T.C.); dingf1991@163.com (F.D.); 2133321@tongji.edu.cn (L.-P.L.); 2133320@tongji.edu.cn (F.C.); 2 Center of Gallbladder Disease, Shanghai East Hospital, School of Medicine, Tongji University, Shanghai 200120, China; 3 Institute of Gallstone Disease, School of Medicine, Tongji University, Shanghai 200331, China

**Keywords:** gallbladder contraction, ultrasound, bile emptying, gallbladder motility disorder, non-invasive treatment

## Abstract

Background: Gallbladder hypomotility is a key pathogenic factor in cholelithiasis. Non-invasive interventions to enhance gallbladder contractility remain limited. Ultrasound therapy has shown promise in various muscular disorders, but its effects on gallbladder function are unexplored. Methods: This study employed low-intensity pulsed ultrasound (LIPUS) at a 3 MHz frequency and 0.8 W/cm^2^ intensity with a 20% duty cycle to irradiate the gallbladder region of fasting guinea pigs. Gallbladder contractile function was evaluated through multiple complementary approaches: in vivo assessment via two-dimensional/three-dimensional ultrasound imaging to monitor volumetric changes; quantitative functional evaluation using nuclear medicine scintigraphy (^99m^Tc-HIDA); and ex vivo experiments including isolated gallbladder muscle strip tension measurements, histopathological analysis, α-smooth muscle actin (α-SMA) immunohistochemistry, and intracellular calcium fluorescence imaging. Results: Ultrasound significantly enhanced gallbladder emptying, evidenced by the volume reduction and increased ejection fraction. Scintigraphy confirmed accelerated bile transport in treated animals. Ex vivo analyses demonstrated augmented contractile force, amplitude, and frequency in ultrasound-treated smooth muscle. Histological examination revealed smooth muscle hypertrophy, α-SMA upregulation, and elevated intracellular calcium levels. Extended ultrasound exposure produced sustained functional improvements without tissue damage. Conclusions: Ultrasound effectively enhances gallbladder contractile function through mechanisms involving smooth muscle structural modification and calcium signaling modulation. These findings establish the experimental foundation for ultrasound as a promising non-invasive therapeutic approach to improve gallbladder motility and potentially prevent gallstone formation.

## 1. Introduction

Approximately one-third of gallstone patients exhibit gallbladder motility disorders, representing a common clinical manifestation of cholelithiasis [1,2]. The diminished contractility of gallbladder smooth muscle is considered a key factor in gallstone formation [3], as the resulting biliary stasis alters bile composition. Elevated cholesterol levels in bile accelerate gallstone formation, as cholesterol more rapidly diffuses into the gallbladder wall, further damaging smooth muscle cells and reducing motility [4,5]. To decrease the occurrence of cholelithiasis and prevent consequences, it is essential to maintain and improve gallbladder mobility as well as stop the· progression of gallstones. For asymptomatic gallstone patients, ursodeoxycholic acid (UDCA) can reduce the concentration of cholesterol and mucin in bile, commonly used for cholesterol gallstones [6]. In conclusion, there is still a lack of effective therapeutic methods that stimulate gallbladder contraction, increase bile expulsion rate, and alleviate the detrimental effects of oversaturated bile on the gallbladder wall.

Low-intensity pulsed ultrasound (LIPUS) is a non-invasive and safe treatment that has been widely accepted as effective [7]. Low-intensity pulsed ultrasound (LIPUS) has been shown to effectively treat various disease models, including fractures, osteoarthritis, and stress urinary incontinence, using energy intensities distinct from those of conventional ultrasound, ranging from 0.01 to 0.1 W/cm^2^ (Ispta) [8,9,10]. Several studies have demonstrated the ability of LIPUS to modulate the differentiation of smooth muscle cells and restore their contractile function, highlighting its substantial therapeutic potential in the management of muscle disorders [9,11]. The biological effects of LIPUS may involve mechanisms such as the regulation of cell proliferation and differentiation, the modulation of inflammatory mediator release, and control of ion signaling channels [12,13]. Previous studies have validated the effective biological effects of LIPUS under different frequency and intensity conditions. Notably, stimulation with LIPUS at a frequency of 3 MHz and intensity of 1 W/cm^2^ significantly promoted cell proliferation [14], while LIPUS at a frequency of 1.15 MHz and intensity of 357 mW/cm^2^ increased cell count and activated motor neurons [13]. Based on previous research findings and the unique properties of gallbladder smooth muscle, we selected a frequency range of 1–3 MHz and an intensity range of 0.3–1.0 W/cm^2^ [15]. This range is particularly relevant as it effectively penetrates to the depth of the gallbladder while activating mechanosensitive calcium channels in smooth muscle tissue, which are critical for the contractile function.

LIPUS may exert specific biological effects on gallbladder smooth muscle by modulating its contraction and enhancing contractile force, thereby influencing overall gallbladder motility [16]. However, there is currently insufficient research available regarding the effects of LIPUS on gallbladder motility. In this study, we utilized LIPUS irradiation on guinea pig gallbladders to modulate gallbladder contraction, aiming to investigate the effects of LIPUS on guinea pig gallbladder emptying and elucidate its potential mechanisms in promoting gallbladder emptying.

To delineate the distinctions between this investigation and our antecedent research, we offer the following elucidation. Our prior work principally examined LIPUS’s capacity to inhibit cholelithiasis by impeding concretion formation in a cholesterol-fed animal model [17]. The present investigation uses hepatobiliary imaging followed by contractility experiments and histological examinations at days 10 and 20 of regular irradiation, where we observed significant changes in gallbladder function and structure [18]. We also concentrate on demonstrating LIPUS’s efficacy in facilitating gallbladder contractility while exploring the fundamental physiological mechanisms underlying this phenomenon.

## 2. Materials and Methods

### 2.1. Animal and Experimental Design

All experiments were approved by the Animal Ethics Board of the University of Tongji Medical School (approval number: TJBB05924Z01; approval date: 24 March 2024). Male guinea pigs (4 weeks old, weight of 250–300 g) were purchased from Shanghai Jiesijie Laboratory Animal Co., Ltd. (Shanghai, China), and housed in standard animal facilities. All laboratory animals were fed standard food and kept in a 12 h light/dark cycle with free access to food and water. After one week of acclimation, the guinea pigs were randomly divided into two groups to compare gallbladder contraction: LIPUS group (*n* = 10): 5 for in vivo and 5 for in vitro experiments; SHAM group (*n* = 10): 5 for in vivo and 5 for in vitro experiments.

To evaluate long-term radiation effects, an additional 9 animals were used. The guinea pigs were randomly divided into three groups (*n* = 3 per group) for 0 days, 10 days, and 20 days of radiation. Half of each gallbladder tissue sample was retained for histological and molecular biology analysis.

### 2.2. Acoustic Field Characterization

The present study employed a flat circular transducer (35 mm diameter, non-focused type, planar wave emission mode) for low-intensity pulsed ultrasound intervention. The transducer was provided by the manufacturer (Model: UT1021, Dongdixin Technology Co., LTD., Shenzhen, China) to produce sinusoidal pulses at a 3 MHz frequency with a 20% duty cycle (2 ms pulse width, 8 ms interval) and a 10 ms pulse repetition period (spatial-average temporal-average intensity (Ispta): 0.8 W/cm^2^; treatment duration: 15 min; duty cycle: 20%; pulse repetition period: 10 ms; diameter: 35 mm; effective radiating area: 9.61 cm^2^). The acoustic parameters were characterized using the far-field sound pressure method. Measurements were performed at a distance of 210 mm from the transducer surface using a calibrated hydrophone. The effective radiating surface area of the transducer was 9.61 cm^2^. In the standard operating conditions (0.8 W/cm^2^), the following parameters were measured:

- Peak-to-peak sound pressure: 0.56 MPa.
- Hydrophone voltage: 624 mVpp.
- Effective radiated acoustic power: 2.67 W.
- Spatially averaged effective intensity: 0.277 W/cm^2^.

For different duty cycle settings, the actual time-averaged intensity (I_a_) was calculated using the following equation:

I_a_ = I × DC

where I is the effective intensity and DC is the duty cycle expressed as a percentage.

### 2.3. LIPUS Radiation

Prior to the experiment, the guinea pigs underwent a 12 h fasting period and received mask inhalation anesthesia with isoflurane (concentration: 1.5% to 2.5%, RWD Life Science, Shenzhen, Guangdong Province, China). The abdominal hair was shaved, and the animals were placed on a heated pad maintained at 35 °C. Due to the variability in gallbladder position caused by individual anatomical differences and respiratory motion, the gallbladder location was determined for each animal prior to LIPUS stimulation. Ultrasound imaging was used to identify the gallbladder and confirm its position relative to the transducer (Supplementary Materials Figure S1). The movement range of the gallbladder boundary was determined under the influence of respiratory motion. After identifying the surface projection area, the ultrasound beam was adjusted to ensure its coverage exceeded the gallbladder’s movement range. Once the gallbladder was located, the transducer was positioned accordingly, and a mechanical holder was used to maintain consistent contact and alignment throughout the 15 min sonication period. Acoustic coupling gel was applied between the transducer and the skin surface to ensure efficient ultrasound transmission. During radiation, the adhesive was replaced promptly to ensure the radiation area temperature remained constant. Upon completion of LIPUS Parameter Optimization, we used an orthogonal experimental design (Supplementary Materials Figures S2–S4). To evaluate long-term radiation effects, long-term radiation groups were included in the LIPUS radiation study for 10 days and 20 days of treatment. The same parameters were used for daily radiation treatments, administered twice a day with an 8 h interval between sessions.

### 2.4. Ultrasound Image

Ultrasound examinations were performed using the Philips Epiq 7 system with GI3DQ software (V6-5), employing both 2D (frequency range: 18.0 to 5.0 MHz, x18-5) and 3D (frequency range: 6.0 to 1.0 MHz, x13-6, ISPTA < 30 mW/cm^2^, MI < 0.7) ultrasound techniques to assess guinea pig gallbladder volume and contractile function. Baseline images were acquired before the initiation of ultrasonication, with images obtained immediately after stimulation and at 15 and 30 min post-stimulation to assess changes in gallbladder contraction. For 2D ultrasound, gallbladder volume changes were determined using the Dodds formula: longitudinal diameter × transverse diameter × anteroposterior diameter × 0.52 [19]. The 3D volume assessment employed the GI3DQ software, which automatically provided volumetric measurements and morphological characteristics through contour-based reconstruction. The gallbladder-emptying fraction (GBEF) at different time points was calculated using the formula: GBEF = [(volume during fasting state − volume during contraction state)/volume during fasting state] × 100.

### 2.5. Nuclear Medicine Image

^99m^Tc-EHIDA hepatobiliary scintigraphy was utilized as the reference standard to assess gallbladder contraction in guinea pigs [20]. Nuclear medicine imaging was performed on 6 guinea pigs (3 from the LIPUS group and 3 from the sham group). The guinea pigs were positioned supine on the scanning platform, and all subjects underwent computed tomography scanning to visualize the gallbladder and exclude gallstones. Subsequently, a radiotracer (0.5 mCi of ^99m^Tc-EHIDA) was administered slowly over 3 min. The ^99m^Tc-EHIDA scan was initiated after the baseline screening scan. Sequential scintigraphy images of the gallbladder were acquired at 2 min intervals until optimal visualization was achieved. Upon achieving optimal visualization, a 15 min stimulation with LIPUS was initiated. Hepatobiliary imaging was continued until the 90th minute to determine the peak ejection fraction (EF), representing the maximum EF value achieved during the imaging period [21,22].

### 2.6. Measurement of Force in Gallbladder Smooth Muscle

After euthanasia (following deep anesthesia with 4% isoflurane, cervical dislocation was performed), the gallbladders of guinea pigs were immediately immersed in Krebs–Henselite (K-H) solution pre-oxygenated with 95% O_2_ and 5% CO_2_ at 4 °C. The K-H solution comprised NaCl (113 mM), KCl (4.7 mM), CaCl_2_ (2.5 mM), KH_2_PO_4_ (1.2 mM), MgSO_4_ (1.2 mM), NaHCO_3_ (25 mM), and glucose (11.5 mM). Smooth muscle force measurements were conducted on 10 samples (5 from the LIPUS group and 5 from the sham group). A ring-shaped muscle strip, 1.0 cm in diameter and 0.3 cm in width, was extracted from the short axis of each gallbladder. The muscle strips were suspended in an organ circulation bath containing K-H solution (pH 7.4, 37 °C), with continuous bubbling of 95% O_2_ and 5% CO_2_ gas. One end of each muscle strip was securely fastened to a hook at the base of the organ bath using surgical silk thread, while the other end was connected to an isometric force transducer. The force transducer was linked to an integrated amplifier and recorder. Each muscle strip sample was subjected to an initial tension of 1.5 g and allowed to equilibrate for 45 min before commencing the experimental procedure. The ultrasound transducer was positioned 8 mm from the immobilized gallbladder tissue. This alignment maintained consistent acoustic coupling across all specimens. Concomitantly, the tension, amplitude, and frequency of muscle contractions were recorded as they varied over time.

In this experiment, acetylcholine (ACh) was used to stimulate gallbladder smooth muscle contraction. ACh binds to muscarinic receptors on smooth muscle cells, triggering intracellular calcium release and activating the contractile machinery [23]. This process mimics physiological conditions and allows us to evaluate the contractile response of gallbladder tissue under LIPUS treatment. ACh at a concentration of 10 μmol/L was administered. Changes in gallbladder tension elicited by ACh were documented while the ACh-induced effects were recorded as the response value.

Tension data were acquired and analyzed using a data collection and analysis system (ADI, New South Wales, Australia). The recorded data were subjected to analysis to determine the rate of change (R), calculated as the absolute difference between the response value and the control value, divided by the control value. The result is expressed as the rate of change (R), where R = [|(Response value − control value)|/Control value].

### 2.7. Histological Analysis

Gallbladders were harvested at specific time points, fixed in 4% formalin, and embedded in paraffin. Paraffin sections (5 μm thick) were dewaxed in xylene and rehydrated with ethanol. Masson’s trichrome staining was performed according to the standard protocol. Five images were randomly taken, and the smooth muscle content in the gallbladder was calculated using Image-J (v 1.53) (Media Cybernetics, Silver Spring, MD, USA).

### 2.8. Immunofluorescence Staining Analysis

Each group of three muscle strips was removed from 4% paraformaldehyde and dehydrated with sucrose solution. After cryosectioning, sections were sealed with 3% BSA for 30 min and incubated with anti-α-SMA (1:1000, My Bio Source, San Diego, CA, USA) to identify smooth muscle cells in the muscle layer. Before adding primary antibodies, non-specific antibody binding was blocked with 3% bovine serum albumin (BSA). The samples were then incubated with an appropriate streptavidin-biotin-horseradish peroxidase (HRP)-bound secondary antibody (Wuhan Saiville Biotechnology Co., Ltd., Wuhan, China). The peroxidase reaction was carried out using H_2_O_2_ and 3,3-diaminobenzidine (DAB) tetrahydrochloride (Wuhan Servicebio Technology Company, Wuhan, China) as chromogenic agents for antibody localization. Nuclei were stained with DAPI (Servicebio, g1012). Images were taken at ×200 magnification under an inverted fluorescence microscope (Olympus Bx-51, Tokyo, Japan). Images were acquired using Caseviewer 2.1, and the cross-sectional area of muscle fibers was calculated using ImageJ. The mean transverse muscle fiber area was calculated as the total cross-sectional area divided by the total number of muscle fibers in the field of view.

### 2.9. Ca^2+^ Image

The guinea pig was euthanized. GBSM cells were enzymatically isolated using the method based on the previously described guinea pig gallbladder technique [24]. Ca^2+^ imaging was performed on 6 samples (3 from the LIPUS group and 3 from the SHAM group). The gallbladder was removed and placed in a solution of cold Krebs–Henseleit (113 mM NaCl, 4.7 mM KCl, 2.5 mM CaCl_2_, 1.2 mM KH_2_PO_4_, 1.2 mM MgSO_4_, 25 mM NaHCO_3_, and 11.5 mM D-glucose). The solution, after equilibrating with 95% CO_2_ and 5% O_2_, reached a final pH of 7.35 in Sylgard-coated culture dishes. After removing the mucosa attached to the gallbladder and the connective tissue on the liver, the gallbladder was cut into small pieces and incubated for 35 min in an enzyme solution at 37 °C (composed of 10 mM HEPES, 55 mM NaCl, 5.6 mM KCl, 80 mM sodium glutamate, 2 mM MgCl_2_, and 10 mM D-glucose, with NaOH adjusting the pH to 7.3), supplemented with 1 mg/mL BSA, 1 mg/mL papain, and 0.5 mg/mL Dithiothreitol (DTT). Then, we transferred the tissue to fresh ES containing 1 mg/mL BSA, 1 mg/mL collagenase, and 100 µM CaCl_2_ and incubated it in this solution at 37 °C for 9 min. After digestion was complete, we washed the tissue three times with ES. The resulting cell suspension was kept in ES at 4 °C, prepared for irradiation.

To optimize the detection of intracellular calcium ion changes, the LIPUS parameters for cell-level experiments were adjusted to an intensity of 0.3 W/cm^2^, an ultrasound frequency of 1.0 MHz, and a duty cycle of 30% with a stimulation duration of 30 s. These parameters differ from the in vivo settings because (1) the lower intensity (0.3 W/cm^2^ vs. 0.8 W/cm^2^) prevents direct cellular damage while still providing sufficient mechanical stimulation; (2) the lower frequency (1.0 MHz vs. 3 MHz) offers better penetration in the cell culture medium with less attenuation; and (3) the slightly higher duty cycle (30% vs. 20%) compensates for the reduced intensity while maintaining adequate energy delivery for calcium channel activation in isolated cells. These modified parameters were selected based on previously established protocols for cellular calcium imaging with ultrasound stimulation [25], which demonstrated optimal cellular response without inducing stress or damage to the isolated cells. Despite these parameter adjustments, the fundamental mechanical stimulation mechanism remains consistent with the in vivo application, enabling valid insights into the calcium signaling response while optimizing the experimental conditions for isolated cells.

The control cells received a sham treatment. The suspension of smooth muscle cells from the gallbladder was added to a six-well plate, and both the irradiated and sham-treated cell samples were incubated with a 4 μM Flou-4 AM solution in 1 mM K-H balanced salt at 37 °C for 30 min. All samples underwent fluorescence calibration using Flou-4 AM, a fluorescent dye that permeates the cell membrane. Flou-4 AM is metabolized by intracellular esterases to form Flou-4, which remains within the cells and binds to calcium ions. Calcium fluorescence, excited at 488 nm, was detected using a fluorescence microscope. The experiment was repeated three times to ensure the reliability of the results.

### 2.10. Protein Extraction and Western Blot Analysis

For protein expression analysis, three gallbladder tissues per group were minced and lysed using radioimmunoprecipitation (RIPA) lysis buffer (Sigma-Aldrich, St. Louis, MO, USA). Equal amounts of protein were then separated by 10% SDS polyacrylamide gel electrophoresis and transferred to a Mozon polyvinylidene difluoride membrane (Merck Millipore, Darmstadt, Germany). Immunoblotting was performed with primary antibodies, including anti-α-SMA (1:500, My Bio Source, San Diego, CA, USA) and anti-α-tubulin (1:500, Invitrogen, Carlsbad, CA, USA), overnight at 4 °C. After washing the membrane with TBST, it was incubated with the appropriate HRP-conjugated secondary antibody for 1 h at room temperature. The ECL Plus Chemiluminescence Kit (Vazyme Biotech, Nanjing, China) was used for visualization of the Western blot, which was subsequently quantified using ImageJ software (ImageJ 1.53, NIH, Bethesda, MD, USA). The results were normalized using α-tubulin as an internal control.

### 2.11. Statistical Analysis

All experiments were repeated at least three times to confirm the reliability of the study. The normality of data distribution was first assessed using the Shapiro–Wilk test. After confirming normal distribution (*p* > 0.05), statistical analyses were performed using a *t*-test (α = 0.05) or a one-way analysis of variance (ANOVA) using GraphPad Prism version 8.0 GraphPad, San Diego, CA, USA. All data were presented as the mean value ± standard deviation (SD). *p* < 0.05 was considered statistically significant.

## 3. Results

### 3.1. Evaluation of Injury in Irradiated Areas

**Figure 1:**
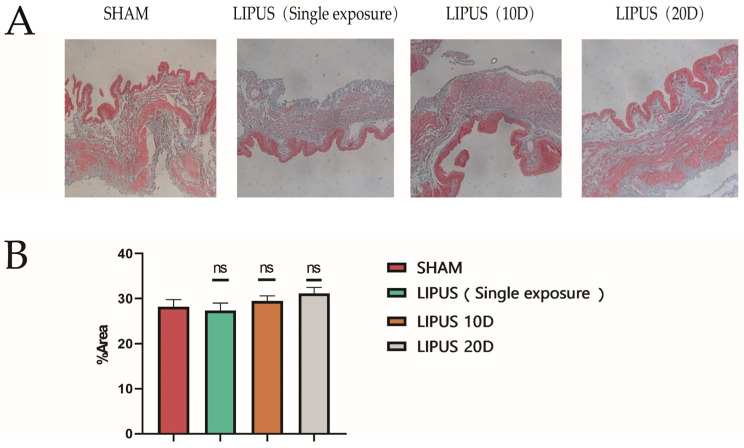
Evaluation of injury in irradiated area. Representative image of Masson staining used to assess LIPUS damage to gallbladder smooth muscle cells. (**A**) Gallbladder Masson staining results (Scale bar 200 µm) in SHAM, LIPUS (Single exposure), LIPUS (10D), and LIPUS (20D) groups; the blue area in the figure is the fibrotic area. (**B**) The graphs show the percentage of fibrotic area. SHAM: sham-irradiated group, the values are the means ± SDs, *p* > 0.05, no significant difference.; LIPUS (Single exposure): LIPUS irradiation once; LIPUS (10D): LIPUS 10-day group; LIPUS (20D): LIPUS 20-day group. Statistical significance is indicated as follows: ns: not significant.

To ensure the safety of the LIPUS application, we implemented comprehensive monitoring protocols throughout the experimental procedures. Temperature sensors were utilized to continuously monitor the surface temperature of the irradiated area in real time, preventing significant thermal effects that could potentially damage gallbladder tissue or cause cutaneous burns in guinea pigs. The ultrasound coupling agent was promptly replaced during treatments to maintain stable interface temperatures and optimal acoustic transmission. Gallbladder tissues from each group of guinea pigs were collected for Masson staining (Figure 1A). In order to assess the biological safety of LIPUS [26,27], there were no significant changes in the proportion of fibrotic areas among the groups throughout the entire experimental period, compared to the SHAM surgery, and there was no obvious difference in the level of fibrosis in the gallbladders (Figure 1B).

### 3.2. The Impact of LIPUS on Gallbladder Contraction

**Figure 2:**
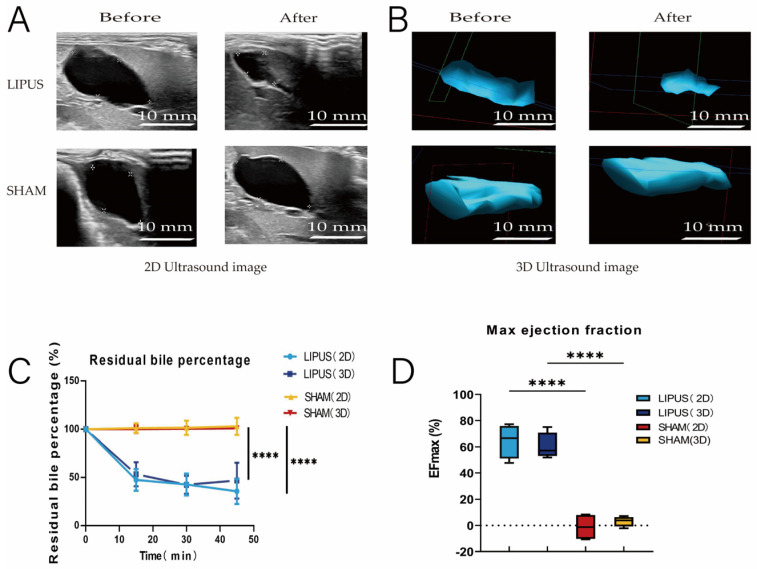
(**A**,**B**) Ultrasound evaluation of pre-irradiation and post-irradiation gallbladder images (left: 2D ultrasound, right: 3D ultrasound). Gallbladder contracted in the LIPUS group and did not contract in the SHAM group. pre: pre-irradiation; post: post-irradiation. (**C**) Trend of gallbladder residual bile percentage over time in SHAM and LIPUS groups (*p* < 0.05). Residual bile percentage: the percentage of remaining gallbladder volume after bile excretion to the original gallbladder volume; (**D**) maximum gallbladder-emptying fraction in SHAM and LIPUS groups, (*p* < 0.05). EFmax: maximum emptying fraction. Statistical significance is indicated as follows: **** *p* < 0.001 compared to control group or as indicated by bars.

To investigate the effect of LIPUS stimulation on the gallbladder, guinea pigs were fasted for 12 h, and their gallbladders were irradiated with LIPUS. Both 2D and 3D ultrasound images demonstrated that the gallbladder in the SHAM group did not shrink significantly, while the gallbladder volume in the LIPUS group changed significantly after irradiation (Figure 2A,B). Compared to the SHAM group, the gallbladder volume in the LIPUS irradiation group decreased significantly within 45 min after stimulation. The percentage of residual bile volume (the ratio of remaining gallbladder volume to the original gallbladder volume after volume change) decreased progressively (2D: 35.53 ± 12.99%; 3D: 46.81 ± 18.53%) (*p* < 0.05) (Figure 2C), and the maximum ejection fraction of the gallbladder showed significant differences (2D: 64.46 ± 12.99%; 3D: 60.31 ± 10.13%) (*p* < 0.05) (Figure 2D). The results indicated that exposure to LIPUS led to a significant reduction in gallbladder volume compared to the SHAM group.

### 3.3. Hepatocholescintigraphy

**Figure 3:**
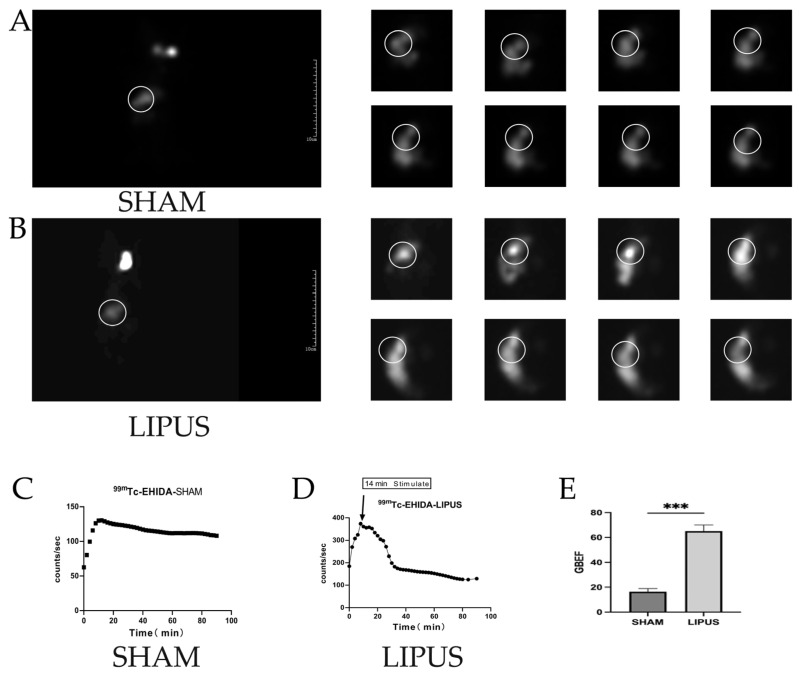
(**A**) Results of nuclear hepatobiliary imaging of the gallbladder of guinea pigs in the SHAM group (0–90 min); (**B**) results of nuclear hepatobiliary imaging of guinea pigs in the LIPUS group (0–90 min). (**C**,**D**) Trends in the radioactivity counts of gallbladder 99mTC-HIDA in the biliary system of the SHAM and LIPUS groups. (**E**) Gallbladder-emptying fraction in the SHAM and LIPUS groups (*p* < 0.05), with statistically significant differences. Circles in the figure highlight the metabolic pathway of bile between the liver and gallbladder. These marked areas emphasize the critical transport routes involved in bile metabolism, secretion, and storage. LIPUS: low-intensity pulsed ultrasound group; SHAM: sham irradiation group. Statistical significance is indicated as follows: *** *p* < 0.001 compared to control group or as indicated by bars.

To further confirm the effect of LIPUS on gallbladder contraction, hepatocholescintigraphy was performed, revealing that the emptying rate of ^99m^Tc-HIDA in the fasting gallbladder was significantly higher in the LIPUS irradiation group compared to the SHAM group. After achieving optimal visualization of the gallbladder, LIPUS irradiation was applied, and continuous recordings of ^99m^Tc-HIDA radioactive counts were taken within 90 min post-irradiation (Figure 3). In the SHAM group, the gallbladder remained filled, displaying slow bile transport (Figure 3A,C). In contrast, the LIPUS irradiation group exhibited rapid emptying behavior (Figure 3B,D), with a gallbladder ejection fraction (GBEF) of 65.11 ± 8.66% (Figure 3E). The significant difference in GBEF between the two groups (*p* < 0.05) confirmed that LIPUS contributes to promoting bile circulation.

### 3.4. Gallbladder Muscle Strip Contraction Experiment

**Figure 4:**
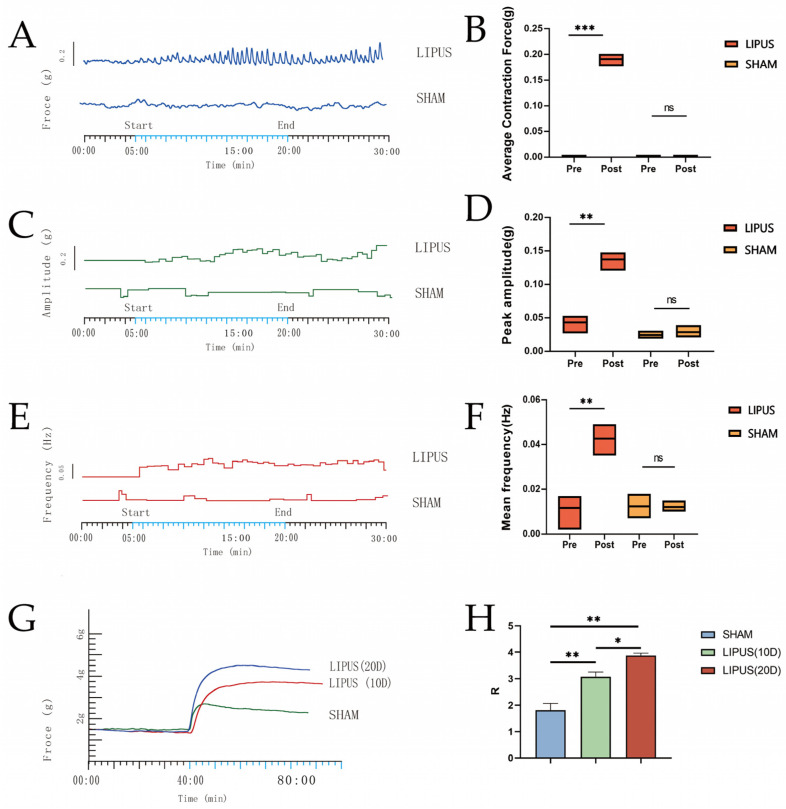
Plots of contraction force (**A**), contraction amplitude (**C**), and contraction frequency (**E**) fluctuations over time in the gallbladder muscle strips of the LIPUS and SHAM groups. (**B**) Average contraction force of gallbladder muscle strips before and after irradiation in the LIPUS and SHAM groups (*p* < 0.05). (**D**) Peak amplitudes of gallbladder muscle strips before and after irradiation in the LIPUS and SHAM groups. (*p* < 0.05). (**F**) Mean frequency of gallbladder muscle strips before and after irradiation in the LIPUS and SHAM groups (*p* < 0.05). (**G**,**H**) Contraction force in SHAM group, LIPUS (10D) group, LIPUS (20D) group; rate of change in muscle contraction tension in each group (*p* < 0.05), R-value: rate of change in tension = [| (response value − control value) |/control value]. Statistical significance is indicated as follows: ns: not significant, * *p* < 0.05, ** *p* < 0.01, *** *p* < 0.001 compared to control group or as indicated by bars.

The muscle tension experiment results demonstrated that after a single LIPUS irradiation (Figure 4), the isolated gallbladder smooth muscle strips exhibited sustained and rhythmic contractions (Figure 4A–C), whereas no significant changes were observed in the SHAM group (Figure 4A–C). Compared to the SHAM group, the LIPUS irradiation group exhibited significant increases in gallbladder smooth muscle contraction tension, contraction amplitude, and contraction frequency, indicating statistical significance (Figure 4D–F). A significant difference was observed in the peak contraction amplitude (0.14 ± 0.01 g) (Figure 3E) of the gallbladder muscle strips before and after LIPUS irradiation in the LIPUS group (*p* < 0.05), while no significant difference was noted in the SHAM group (*p* > 0.05). Additionally (Figure 4F), the average contraction frequency (0.04 ± 0.01 Hz) of the gallbladder muscle strips before and after LIPUS irradiation in the LIPUS group also showed a significant difference (*p* < 0.05), with no significant difference observed in the SHAM group (*p* > 0.05).

As shown in (Figure 4G,H), after receiving LIPUS irradiation for 10 days (10D) and 20 days (20D), respectively, and following the induction of contraction in isolated gallbladder smooth muscle with the same concentration of ACh, the contraction force of the gallbladder smooth muscle was lowest in the SHAM group. The muscle contraction R values for LIPUS (10D) (3.08 ± 0.32) and LIPUS (20D) (3.87 ± 0.15) were significantly higher than that of SHAM (1.81 ± 0.45) (*p* < 0.05), and the muscle contraction R value for LIPUS (20D) was notably higher than that for LIPUS (10D). The results of ACh-induced contraction prove that the contraction force of the gallbladder was directly proportional to the number of irradiations.

### 3.5. LIPUS Affects Gallbladder Smooth Muscle Layer

**Figure 5:**
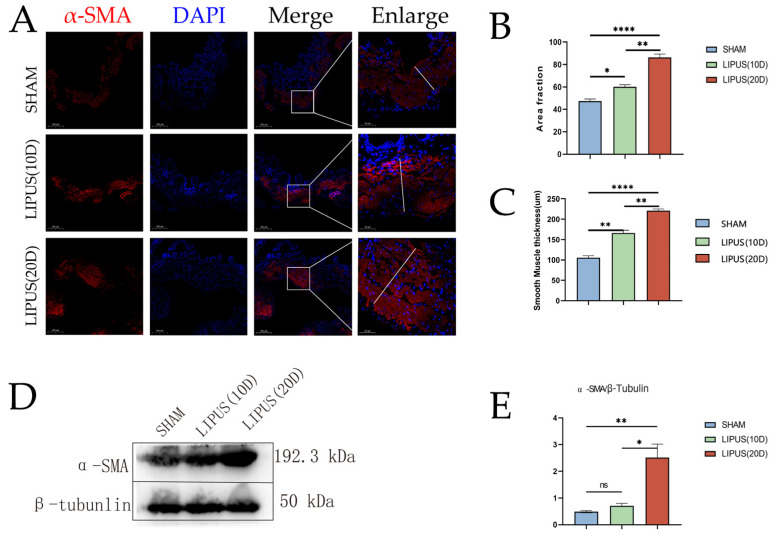
Immunofluorescence staining of smooth muscle cells with antibodies against α-SMA (red). Nuclei were stained with DAPI (blue) to visualize the nuclear translocation of α-SMA in gallbladder smooth muscle cells. Scale bar = 200 µm. (**A**) Immunofluorescence staining results of smooth muscle actin (α-SMA) in SHAM group, LIPUS (10D) group, and LIPUS (20D) group. Scale bar length is 200 µm. (**B**) Percentage of positive staining for smooth muscle actin (α-SMA) in the SHAM group, LIPUS (10D) group, and LIPUS (20D) group (*p* < 0.05). (**C**) Smooth muscle layer thickness of the SHAM group, LIPUS (10D) group, and LIPUS (20D) group (*p* < 0.05). (**D**,**E**) Results of immunoblot analysis of smooth muscle actin (α-SMA) in SHAM group, LIPUS (10D) group, and LIPUS (20D) group. Protein band density (relative to β-tubunlin) of α-SMA (*p* < 0.05). Statistical significance is indicated as follows: ns: not significant, * *p* < 0.05, ** *p* < 0.01, **** *p* < 0.001 compared to control group or as indicated by bars.

Immunofluorescence staining and immunoblot analysis were performed to determine the effect of LIPUS irradiation on the gallbladder smooth muscle marker α-SMA (Figure 5). The immunofluorescence staining results showed that the percentage of smooth muscle actin (α-SMA) positive staining was higher in the LIPUS (20D) group (86.48 ± 4.65) compared to the SHAM group (47.26 ± 3.32). The difference in the SMA-positive staining percentage between the LIPUS irradiation group and the SHAM group was statistically significant (*p* < 0.05) (Figure 5), indicating an increase in the proportion of the muscle layer area. The immunoblot analysis further confirmed the differential expression of α-SMA in the gallbladder muscle layer, with the SHAM group (0.49 ± 0.07) and the LIPUS (20D) group (2.51 ± 0.88) demonstrating the impact of LIPUS on the protein content of α-SMA in the gallbladder muscle layer. Compared to the SHAM group, the expression level of α-SMA significantly increased after 20 days of LIPUS irradiation (*p* < 0.05). The protein levels of smooth muscle significantly increased after irradiation (Figure 1). In summary, these findings suggest that the enhancement of smooth muscle function may be related to gallbladder contraction. Based on these findings, we conclude that LIPUS irradiation promotes the thickening of the smooth muscle layer in the gallbladder, thereby enhancing its contractile ability. These effects may have significant implications for the contraction activity of the gallbladder.

### 3.6. Calcium Ion Fluorescence Intensity in Gallbladder Smooth Muscle Cells

**Figure 6:**
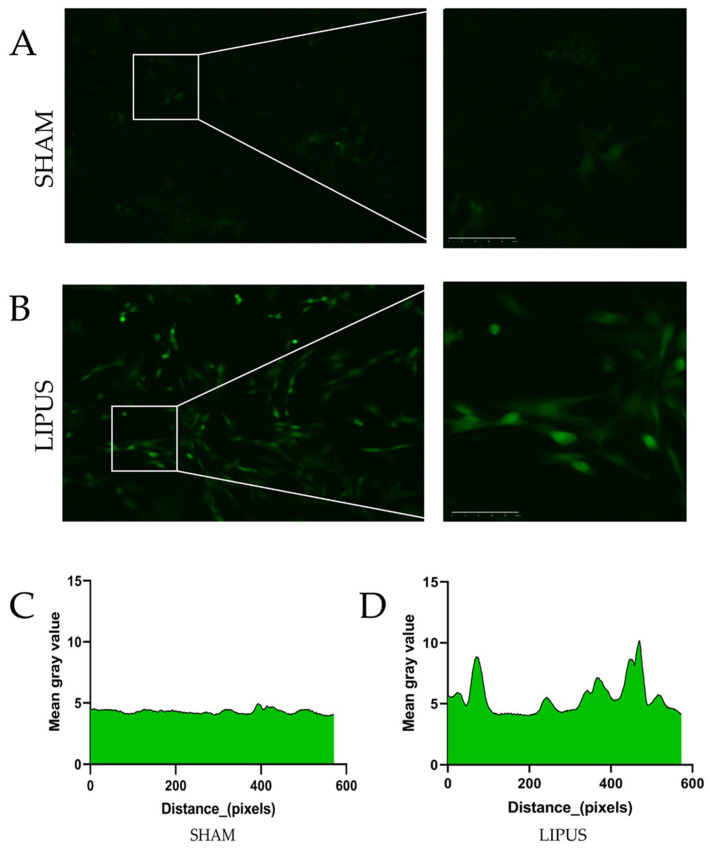
The response of gallbladder smooth muscle cells to LIPUS stimulation. (**A**) Representative fluorescence images of gallbladder smooth muscle cells in the SHAM group showing minimal changes in calcium ion fluorescence intensity over time. (**B**) Cells in the LIPUS group demonstrating marked increase in fluorescence intensity following ultrasound stimulation (1 MHz, 300 mW/cm^2^, 30% duty cycle). (**C**) Line scan analysis of calcium ion fluorescence intensity along randomly selected regions of interest in SHAM group cells. (**D**) Corresponding analysis in LIPUS-treated cells showing significant elevation in fluorescence intensity, indicating increased cytosolic calcium concentration. Images were acquired using an LSM 880 confocal microscope (Zeiss, Jena, Germany) with 488 nm excitation and 500–550 nm emission. Fluorescence was recorded at 5 s intervals for 5 min. All values were normalized to baseline measurements obtained prior to stimulation. Fluorescence intensity was measured by randomly selecting standardized unit areas and quantifying absolute grayscale values within these regions using ImageJ software (NIH, USA). Scale bar = 20 µm.

To further elucidate the molecular mechanisms underlying LIPUS-induced gallbladder contraction, we conducted irradiation experiments on isolated gallbladder smooth muscle cells (Figure 6). As shown in Figure 6A, the SHAM group exhibited no notable changes in calcium ion fluorescence intensity within smooth muscle cells. In contrast, Figure 6B demonstrates a significant increase in intracellular calcium ion fluorescence intensity following LIPUS stimulation. Quantitative analysis of calcium ion fluorescence intensity along randomly selected pixel distances is presented in Figure 6C,D. These results clearly demonstrate that LIPUS stimulation significantly alters calcium ion dynamics in gallbladder smooth muscle cells, strongly suggesting that increased calcium influx is a key mechanism responsible for the enhanced contractile response observed in our experiments.

## 4. Discussion

In this study, we demonstrated for the first time that LIPUS effectively facilitates gallbladder emptying and smooth muscle contraction in guinea pigs. Through ultrasound observation and hepatobiliary imaging, followed by contractility experiments and histological examinations at days 10 and 20 of regular irradiation, we observed significant changes in gallbladder function and structure. The previous study [17] used a cholesterol-fed guinea pig model to examine gallstone formation, thereby focusing on a preventive approach. The present study employed healthy guinea pig models to investigate gallbladder motility and contraction dynamics. This shift emphasizes the therapeutic potential of LIPUS in treating gallbladder dysmotility, which has broader clinical implications.

Our findings revealed that LIPUS irradiation induced immediate bile emptying behavior, with the contractile force significantly increasing following regular treatment. Isolated gallbladder tissues exhibited enhanced rhythmic variations in contractile force, with significant increases in strength, amplitude, and frequency post-LIPUS exposure. These mechanical changes were supported by histological analysis, which demonstrated thickening of the gallbladder smooth muscle layer and increased α-SMA expression, particularly evident after 20 days of treatment. Many studies have proven that calcium influx contributes to smooth muscle contractility [28,29]. At the cellular level, our calcium fluorescence imaging results demonstrate that LIPUS stimulation leads to increased intracellular calcium concentration in gallbladder smooth muscle cells, consistent with previous research establishing the pivotal role of calcium influx in smooth muscle contraction [16,25,30]. The increased intracellular calcium following LIPUS exposure may represent a key mechanism underlying the enhanced contractile response. This study confirms the effectiveness of LIPUS in enhancing gallbladder contraction, validating its beneficial biological effects in promoting gallbladder contractility in guinea pigs. The demonstrated efficacy in non-diseased tissue suggests broader applications for motility disorders beyond stone prevention.

The gallbladder wall contains muscle bundles arranged in a loose configuration, which permits coordinated contractile and relaxation movements necessary for appropriate emptying and refilling functions [31,32,33]. Gallbladder motility disorders disrupt this coordinated process, leading to abnormal bile flow and disease progression [34,35]. The specific biophysical cascade linking ultrasound exposure to enhanced gallbladder contractility likely involves several interconnected mechanisms [25]. LIPUS stimulation initiates mechanical forces that activate mechanosensitive channels in the smooth muscle cell membrane, particularly stretch-activated calcium channels. This mechanical activation triggers calcium influx through both voltage-dependent L-type calcium channels and receptor-operated calcium channels. The increased intracellular calcium concentration then activates the calcium–calmodulin complex, which subsequently phosphorylates the myosin light chain kinase (MLCK). Activated MLCK phosphorylates myosin light chains enable actin-myosin cross-bridge formation and smooth muscle contraction [36]. Therefore, enhancing gallbladder contraction is crucial in preventing and treating gallbladder diseases. However, there is currently a lack of research focused on effectively improving gallbladder contraction and halting early disease development.

Radionuclide hepatobiliary imaging, using ^99m^Tc-HIDA, is a key tool for assessing gallbladder ejection fraction but lacks standardization [37,38]. Some scholars suggest combining and comparing the two techniques of liver and gallbladder imaging and ultrasound [39]. In this study, LIPUS irradiation significantly reduced gallbladder volume, decreased residual bile volume, and increased the maximum ejection fraction in guinea pigs. Nuclear scintigraphy further confirmed that LIPUS accelerated bile transport, providing further evidence of LIPUS’s ability to promote fasting gallbladder emptying.

LIPUS is a low-intensity, non-invasive technique that exerts biological effects through non-thermal mechanisms like cavitation and acoustic streaming [40]. It stimulates cells to transduce mechanical signals into biochemical responses, triggering downstream pathways [41]. Studies have shown that LIPUS enhances muscle cell proliferation, differentiation, and regeneration, increases muscle fiber diameter, and improves muscle strength and contraction rhythm [42,43,44,45,46,47]. In summary, numerous studies have demonstrated significant changes in muscle contraction rhythm, muscle strength, muscle mass, and muscle fiber cross-sectional area following LIPUS irradiation. Building on these established effects on muscle tissue, our study found that LIPUS stimulation significantly enhanced the contractile force, amplitude, and frequency of isolated gallbladder smooth muscle strips, inducing continuous and rhythmic tension after a single exposure. Cellular experiments revealed a marked increase in Ca^2+^ levels following LIPUS, suggesting enhanced smooth muscle excitability. Furthermore, after 20 days of LIPUS irradiation, gallbladder contractile tension showed sustained improvement, indicating long-term benefits for smooth muscle function.

Beyond the immediate effects on muscle contraction, long-term LIPUS irradiation induced significant structural changes in the gallbladder smooth muscle layer. Histological analysis revealed increased muscle thickness and elevated α-SMA protein expression, a key smooth muscle marker. These findings align with the enhanced contractile force observed in vitro and further validate LIPUS’s ability to augment smooth muscle contractility [48]. While the underlying mechanisms require further investigation, these results establish a theoretical foundation for LIPUS as a promising non-invasive approach to promote gallbladder emptying and facilitate clinical translation.

The acoustic parameters selected for this study (3 MHz, 0.8 W/cm^2^ Ispta) were based on theoretical considerations and preliminary testing, specifically optimized for gallbladder smooth muscle stimulation [15,49]. The 3 MHz frequency was chosen to achieve an optimal balance between tissue penetration and focused energy delivery to the gallbladder. This frequency is higher than commonly used LIPUS applications for bone (typically 1–1.5 MHz) to provide more localized energy deposition in soft tissue structures. The intensity of 0.8 W/cm^2^ is higher than typical bone healing applications (0.03–0.1 W/cm^2^) but remains below the thermal threshold, creating sufficient mechanical stimulation to activate mechanosensitive pathways in smooth muscle while avoiding thermal damage. Our pulse parameters (20% duty cycle, 10ms pulse repetition period) were designed to optimize mechanotransduction effects while minimizing potential adaptation or desensitization of smooth muscle tissue to continuous stimulation.

While our findings demonstrate efficacy and safety in guinea pigs, several considerations are important for potential human translation. The human gallbladder is situated deeper within the abdominal cavity compared to guinea pigs, necessitating adjustments to the ultrasound parameters to ensure adequate penetration while maintaining focused energy delivery to the target. Human tissue shows different acoustic attenuation properties, which would require the calibration of treatment parameters. The larger and more complex human anatomical setting would benefit from imaging-guided targeting strategies to precisely locate and treat the gallbladder, potentially using diagnostic ultrasound for real-time visualization. Additionally, the thicker abdominal wall in humans might necessitate longer treatment durations or modified acoustic parameters to achieve comparable biological effects. Clinical implementations would also need to consider individual variations in gallbladder position, size, and surrounding structures, potentially requiring personalized treatment protocols based on pre-treatment imaging.

There are still some limitations to the present study. Firstly, the study only preliminarily explored the factors influencing gallbladder smooth muscle contraction by LIPUS. However, we believe that this study at least reflects the trend of changes in histology and molecular biology within the gallbladder. Secondly, our study found that calcium ion fluorescence intensity within the gallbladder smooth muscle cells exhibited differential levels after LIPUS irradiation. However, these results are primarily based on fluorescence images, making it difficult to elucidate the specific roles of these ion channels in gallbladder contraction. In the future, single-cell RNA sequencing of conditional knockout mice could play a crucial role in studying the functions of specific receptors or ion channels within particular cells. Additionally, analyzing the interactions between immune cells, stromal cells, and smooth muscle cells and investigating specific functional complexes through cellular communication are crucial experimental approaches to verify the role of LIPUS in promoting gallbladder contraction. This study mainly focuses on our finding that LIPUS irradiation can effectively promote the physiological process of gallbladder emptying. It also investigates the effects of LIPUS on smooth muscle contraction force, histological changes, and calcium fluorescence changes in smooth muscle cells. However, how these changes occur requires further investigation. The current acoustic measurements were limited by basic equipment capabilities. Future research should incorporate professional acoustic field measurement equipment, conduct detailed acoustic characterization, establish standardized calibration procedures, and follow ITRUSST guidelines for standardized reporting.

## 5. Conclusions

This research indicates that low-intensity pulsed ultrasound effectively promotes gallbladder contraction and confers enduring benefits. Heightened smooth muscle contractility and increased calcium ion influx appear crucial for LIPUS-mediated gallbladder emptying. Although further corroboration is needed, these findings underscore the importance of our research and highlight LIPUS as a promising strategy for clinical prevention and treatment. Future research directions should include testing in larger animal models to better approximate human anatomy, the optimization of LIPUS parameters for maximum efficacy and safety, and the identification of specific patient populations who might most benefit from this non-invasive therapy.

## Data Availability

The data from the current study are available from the corresponding author upon reasonable request. Data available on request due to restrictions: The data associated with this research is currently under patent application protection. We are in the process of securing intellectual property rights for the novel methods, materials, and/or technologies described in this work. Therefore, the complete dataset cannot be publicly shared at this time to safeguard the patent application process. Interested researchers may contact the corresponding author with specific data requests, which will be considered on a case-by-case basis in accordance with our intellectual property protection protocol and pending patent applications.

## Supplementary Materials

The following supporting information can be downloaded at: https://www.mdpi.com/article/10.3390/bioengineering12070716/s1, Figure S1: Experimental Setup Documentation; Figure S2: Factor levels in orthogonal experimental design for LIPUS parameter optimization; Figure S3: L12(4^1^ × 2^1^ × 3^1^) orthogonal experimental design matrix; Figure S4: comparing the gallbladder contractile

## References

[1] van Erpecum K.J., van Berge Henegouwen G.P., Stolk M.F., Hopman W.P., Jansen J.B., Lamers C.B. (1992). Fasting gallbladder volume, postprandial emptying and cholecystokinin release in gallstone patients and normal subjects. J. Hepatol..

[2] Patel H., Jepsen J. (2024). Gallstone Disease: Common Questions and Answers. Am. Fam. Physician.

[3] Lavoie B., Nausch B., Zane E.A., Leonard M.R., Balemba O.B., Bartoo A.C., Wilcox R., Nelson M.T., Carey M.C., Mawe G.M. (2012). Disruption of gallbladder smooth muscle function is an early feature in the development of cholesterol gallstone disease. Neurogastroenterol. Motil..

[4] Colecchia A., Sandri L., Bacchi-Reggiani M.L., Portincasa P., Palasciano G., Mazzella G., Roda E., Festi D. (2006). Is it possible to predict the clinical course of gallstone disease? Usefulness of gallbladder motility evaluation in a clinical setting. Am. J. Gastroenterol..

[5] Tong K., Jing C., Wang T., Liu K., Guo W., Zhang Z. (2024). The roles of metal ions in gallstones formation. Asian J. Surg..

[6] Gutt C., Schläfer S., Lammert F. (2020). The Treatment of Gallstone Disease. Dtsch. Arztebl. Int..

[7] Xin Z., Lin G., Lei H., Lue T.F., Guo Y. (2016). Clinical applications of low-intensity pulsed ultrasound and its potential role in urology. Transl. Androl. Urol..

[8] Kim E.D., Won Y.H., Park S.H., Seo J.H., Kim D.S., Ko M.H., Kim G.W. (2019). Efficacy and Safety of a Stimulator Using Low-Intensity Pulsed Ultrasound Combined with Transcutaneous Electrical Nerve Stimulation in Patients with Painful Knee Osteoarthritis. Pain Res. Manag..

[9] Ren Y., Zhu Y., Liu L., Yu T., Dong X. (2016). Ultrasound induces contraction of the bladder smooth muscle. Int. Urol. Nephrol..

[10] Tang L., Wu T., Zhou Y., Zhong Y., Sun L., Guo J., Fan X., Ta D. (2022). Study on synergistic effects of carboxymethyl cellulose and LIPUS for bone tissue engineering. Carbohydr. Polym..

[11] Sun L., An S., Zhang Z., Zhou Y., Yu Y., Ma Z., Fan X., Tang L., Guo J. (2021). Molecular and Metabolic Mechanism of Low-Intensity Pulsed Ultrasound Improving Muscle Atrophy in Hindlimb Unloading Rats. Int. J. Mol. Sci..

[12] Li X., Zhong Y., Zhou W., Song Y., Li W., Jin Q., Gao T., Zhang L., Xie M. (2023). Low-intensity pulsed ultrasound (LIPUS) enhances the anti-inflammatory effects of bone marrow mesenchymal stem cells (BMSCs)-derived extracellular vesicles. Cell. Mol. Biol. Lett..

[13] Truong T.T., Chiu W.T., Lai Y.S., Huang H., Jiang X., Huang C.C. (2022). Ca^2+^ signaling-mediated low-intensity pulsed ultrasound-induced proliferation and activation of motor neuron cells. Ultrasonics.

[14] Salgarella A.R., Cafarelli A., Ricotti L., Capineri L., Dario P., Menciassi A. (2017). Optimal Ultrasound Exposure Conditions for Maximizing C2C12 Muscle Cell Proliferation and Differentiation. Ultrasound Med. Biol..

[15] He Z., Liu Q., Yang R., Zhou Y., Liu X., Deng H., Cong H., Liu Y., Liao L. (2025). Low-Intensity Ultrasound Tibial Nerve Stimulation Suppresses Bladder Activity in Rats. Neuromodulation.

[16] Han N., Cheng S., Jin Y., Li G., Wang H., Jin L. (2024). Low-intensity pulsed ultrasound combined with ST36 modulate gastric smooth muscle contractile marker expression via RhoA/Rock and MALAT1/miR-449a/DLL1 signaling in diabetic rats. Neurogastroenterol. Motil..

[17] Chen F., Guo R., Chen T., Liu L., Ding F., Zhao G., Zhang B. (2025). The Therapeutic Potential of Low-Intensity Pulsed Ultrasound in Enhancing Gallbladder Function and Reducing Inflammation in Cholesterol Gallstone Disease. Bioengineering.

[18] Rai M., Paudel N., Sakhrie M., Gemmati D., Khan I.A., Tisato V., Kanase A., Schulz A., Singh A.V. (2023). Perspective on Quantitative Structure–Toxicity Relationship (QSTR) Models to Predict Hepatic Biotransformation of Xenobiotics. Livers.

[19] Cay A., Imamoğlu M., Sarihan H., Ahmetoğlu A. (2006). Ultrasonographic evaluation of fatty meal stimulated gallbladder contraction in the diagnosis of biliary dyskinesia in children. Acta Paediatr..

[20] Ziessman H.A. (2014). Hepatobiliary scintigraphy in 2014. J. Nucl. Med..

[21] Tseng J., Chen Y., McDonald C. (2024). Biliary Dyskinesia and Hyperkinesis. Surg. Clin. N. Am..

[22] Das S., Lal S.B., Venkatesh V., Bhattacharya A., Saxena A., Thapa B.R., Rana S.V. (2021). Gallbladder motility in children with celiac disease before and after gluten-free diet. Ann. Gastroenterol..

[23] Jie H.W., Jie W., Jianxiong M., Xin Z., Runnan X., Yijia F., Bodong L., Jie H. (2024). Mechanism of denervation muscle atrophy mediated by Ach/p38/MAPK pathway in rats with erectile dysfunction caused by nerve injury. Exp. Cell Res..

[24] Petkov G.V., Balemba O.B., Nelson M.T., Mawe G.M. (2005). Identification of a spontaneously active, Na^+^-permeable channel in guinea pig gallbladder smooth muscle. Am. J. Physiol. Gastrointest. Liver Physiol..

[25] Zhang L., Liu X., Gao L., Ji Y., Wang L., Zhang C., Dai L., Liu J., Ji Z. (2020). Activation of Piezo1 by ultrasonic stimulation and its effect on the permeability of human umbilical vein endothelial cells. Biomed. Pharmacother..

[26] Rompe J.D., Kirkpatrick C.J., Küllmer K., Schwitalle M., Krischek O. (1998). Dose-related effects of shock waves on rabbit tendo Achillis. A sonographic and histological study. J. Bone Jt. Surg. Br..

[27] Wang J., Ren L., Liu X., Xu W., Liu M., Hu P., Wang T., Liu J., Ling Q. (2023). Transcriptomics Reveals Molecular Features of the Bilateral Pelvic Nerve Injury Rat Model of Detrusor Underactivity. Biomolecules.

[28] Burks S.R., Lorsung R.M., Nagle M.E., Tu T.W., Frank J.A. (2019). Focused ultrasound activates voltage-gated calcium channels through depolarizing TRPC1 sodium currents in kidney and skeletal muscle. Theranostics.

[29] Zhong X., Fu J., Song K., Xue N., Gong R., Sun D., Luo H., He W., Pan X., Shen B. (2016). The role of TRPP2 in agonist-induced gallbladder smooth muscle contraction. Sci. China Life Sci..

[30] Przystupski D., Ussowicz M. (2022). Landscape of Cellular Bioeffects Triggered by Ultrasound-Induced Sonoporation. Int. J. Mol. Sci..

[31] Zhong X., Wu F., Gao W., Hu J., Shen B., Zhong K., Peng J., Zhang C., Zhang C. (2024). Effects of Extracellular Matrix Changes Induced by a High-Fat Diet on Gallbladder Smooth Muscle Dysfunction. FBL.

[32] Keshavarz M., Ruppert A.L., Meiners M., Poharkar K., Liu S., Mahmoud W., Winterberg S., Hartmann P., Mermer P., Perniss A. (2024). Bitter tastants relax the mouse gallbladder smooth muscle independent of signaling through tuft cells and bitter taste receptors. Sci. Rep..

[33] Housset C., Chretien Y., Debray D., Chignard N. (2016). Functions of the Gallbladder. Compr. Physiol..

[34] Portincasa P., Di Ciaula A., Vendemiale G., Palmieri V., Moschetta A., Vanberge-Henegouwen G.P., Palasciano G. (2000). Gallbladder motility and cholesterol crystallization in bile from patients with pigment and cholesterol gallstones. Eur. J. Clin. Investig..

[35] Yi S.Q., Ohta T., Tsuchida A., Terayama H., Naito M., Li J., Wang H.X., Yi N., Tanaka S., Itoh M. (2007). Surgical anatomy of innervation of the gallbladder in humans and Suncus murinus with special reference to morphological understanding of gallstone formation after gastrectomy. World J. Gastroenterol..

[36] Fang X., Bogdanov V., Davis J.P., Kekenes-Huskey P.M. (2023). Molecular Insights into the MLCK Activation by CaM. J. Chem. Inf. Model..

[37] Rastogi A., Slivka A., Moser A.J., Wald A. (2005). Controversies concerning pathophysiology and management of acalculous biliary-type abdominal pain. Dig. Dis. Sci..

[38] Morris-Stiff G., Falk G., Kraynak L., Rosenblatt S. (2011). The cholecystokin provocation HIDA test: Recreation of symptoms is superior to ejection fraction in predicting medium-term outcomes. J. Gastrointest. Surg..

[39] Kaoutzanis C., Davies E., Leichtle S.W., Welch K.B., Winter S., Lampman R.M., Arneson W. (2014). Abdominal ultrasound versus hepato-imino diacetic acid scan in diagnosing acute cholecystitis—What is the real benefit?. J. Surg. Res..

[40] Xu M., Wang L., Wu S., Dong Y., Chen X., Wang S., Li X., Zou C. (2021). Review on experimental study and clinical application of low-intensity pulsed ultrasound in inflammation. Quant. Imaging Med. Surg..

[41] Wozniak M.A., Chen C.S. (2009). Mechanotransduction in development: A growing role for contractility. Nat. Rev. Mol. Cell Biol..

[42] Fleischman A., Vecchio C., Sunny Y., Bawiec C.R., Lewin P.A., Kresh J.Y., Kohut A.R. (2015). Ultrasound-induced modulation of cardiac rhythm in neonatal rat ventricular cardiomyocytes. J. Appl. Physiol..

[43] Gwak S.J., Bhang S.H., Kim I.K., Kim S.S., Cho S.W., Jeon O., Yoo K.J., Putnam A.J., Kim B.S. (2008). The effect of cyclic strain on embryonic stem cell-derived cardiomyocytes. Biomaterials.

[44] Kada K., Yasui K., Naruse K., Kamiya K., Kodama I., Toyama J. (1999). Orientation change of cardiocytes induced by cyclic stretch stimulation: Time dependency and involvement of protein kinases. J. Mol. Cell. Cardiol..

[45] Sun Z. (2021). Low intensity pulsed ultrasound information technology intervention in diagnosis and prediction of Muscle Atrophy. Pak. J. Med. Sci..

[46] Lei H., Xin H., Guan R., Xu Y., Li H., Tian W., Wang L., Gao Z., Guo Y., Lue T.F. (2015). Low-intensity Pulsed Ultrasound Improves Erectile Function in Streptozotocin-induced Type I Diabetic Rats. Urology.

[47] Qin H., Luo Z., Sun Y., He Z., Qi B., Chen Y., Wang J., Li C., Lin W., Han Z. (2023). Low-intensity pulsed ultrasound promotes skeletal muscle regeneration via modulating the inflammatory immune microenvironment. Int. J. Biol. Sci..

[48] Duan H., Chen S., Mai X., Fu L., Huang L., Xiao L., Liao M., Chen H., Liu G., Xie L. (2024). Low-intensity pulsed ultrasound (LIPUS) promotes skeletal muscle regeneration by regulating PGC-1α/AMPK/GLUT4 pathways in satellite cells/myoblasts. Cell. Signal..

[49] Marcotulli M., Barbetta A., Scarpa E., Bini F., Marinozzi F., Ruocco G., Casciola C.M., Scognamiglio C., Carugo D., Cidonio G. (2024). Jingle Cell Rock: Steering Cellular Activity With Low-Intensity Pulsed Ultrasound (LIPUS) to Engineer Functional Tissues in Regenerative Medicine. Ultrasound Med. Biol..

