# Supplementary figures and images for: Ultrasound Improves Gallbladder Contraction Function: A Non-Invasive Experimental Validation Using Small Animals
Source: Bioengineering (Basel). 2025 Jun 30;12(7):716. doi: 10.3390/bioengineering12070716 (PMC12292603; doi:10.3390/bioengineering12070716)

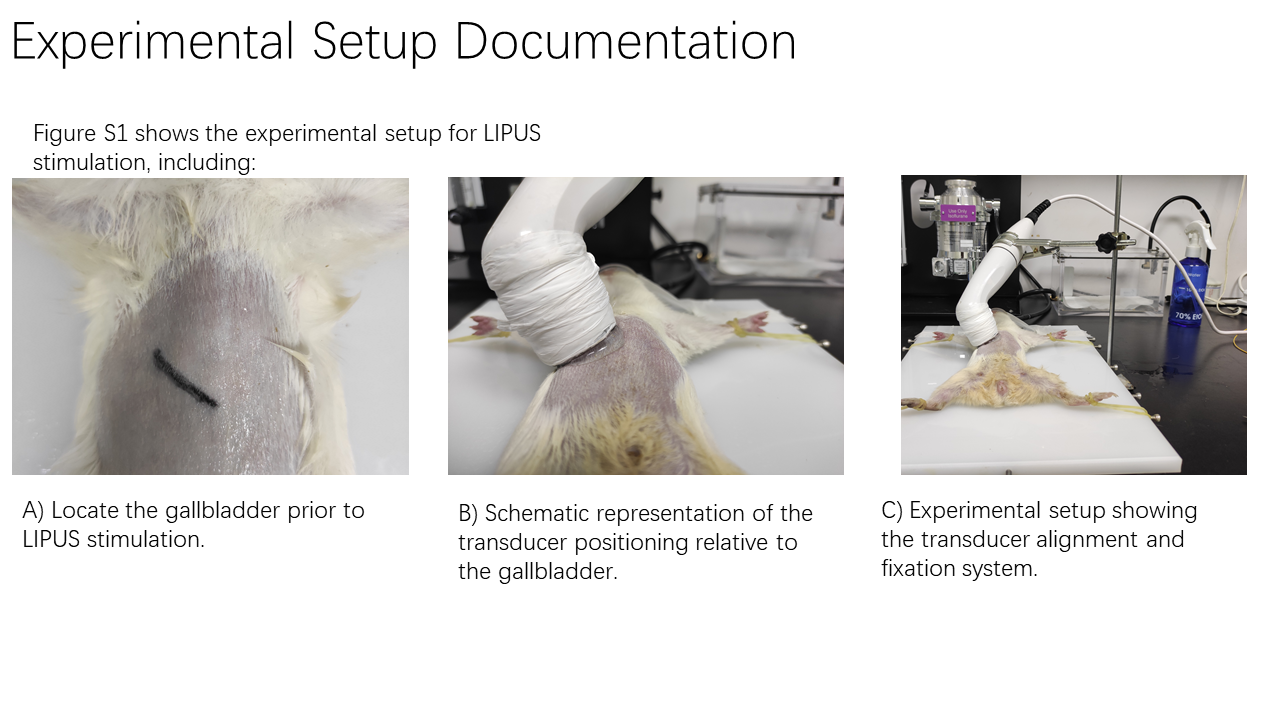

Supplement: Supplementary file 1 [file bioengineering-12-00716-s001.zip › S1.tif]

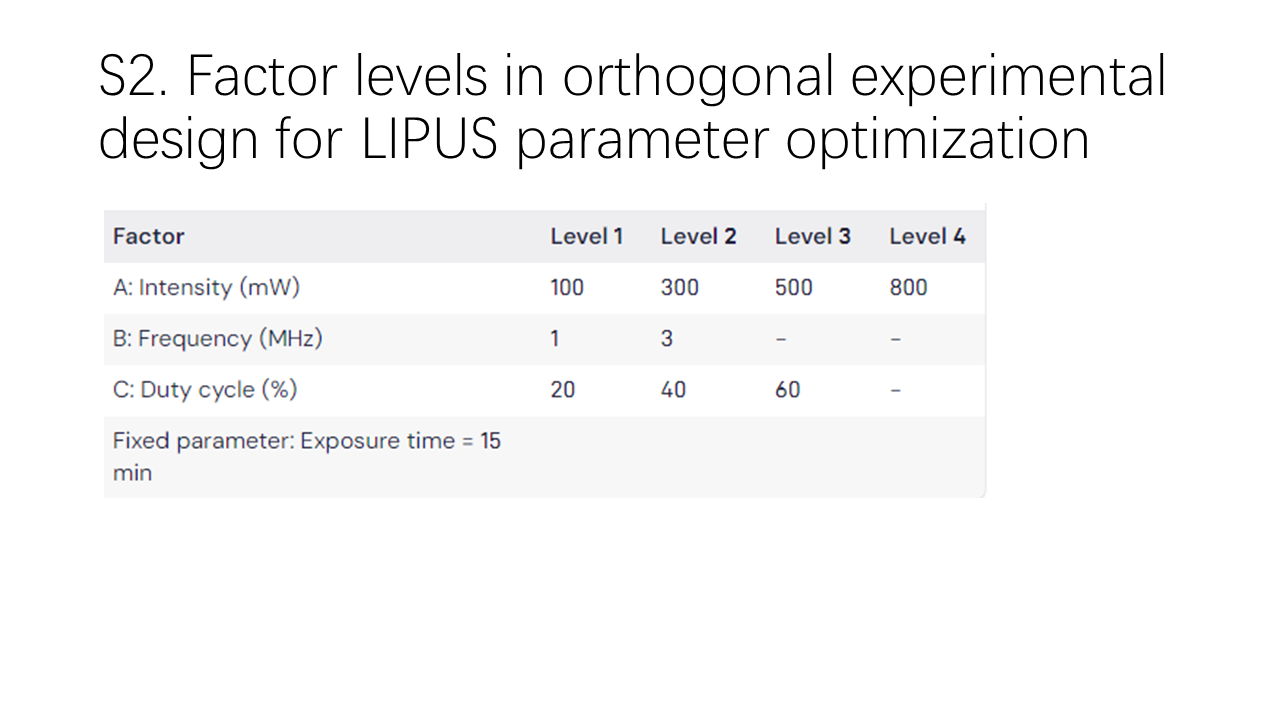

Supplement: Supplementary file 1 [file bioengineering-12-00716-s001.zip › S2.tif]

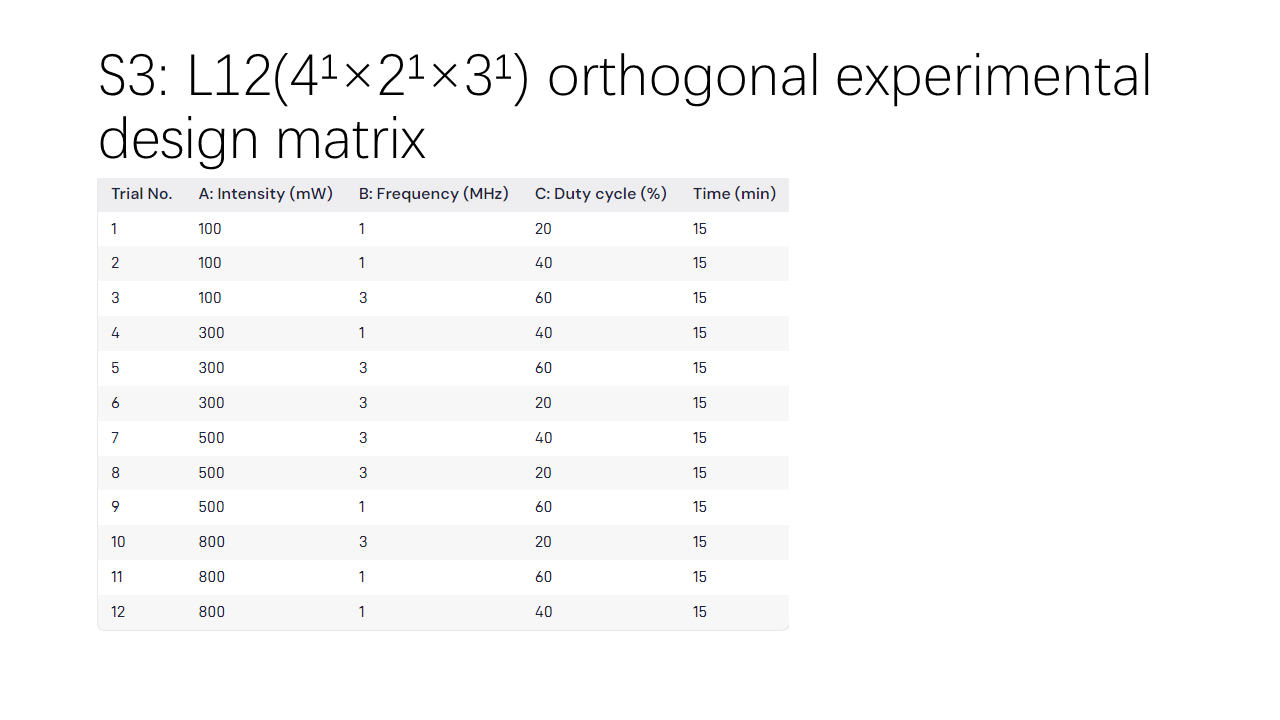

Supplement: Supplementary file 1 [file bioengineering-12-00716-s001.zip › S3.tif]

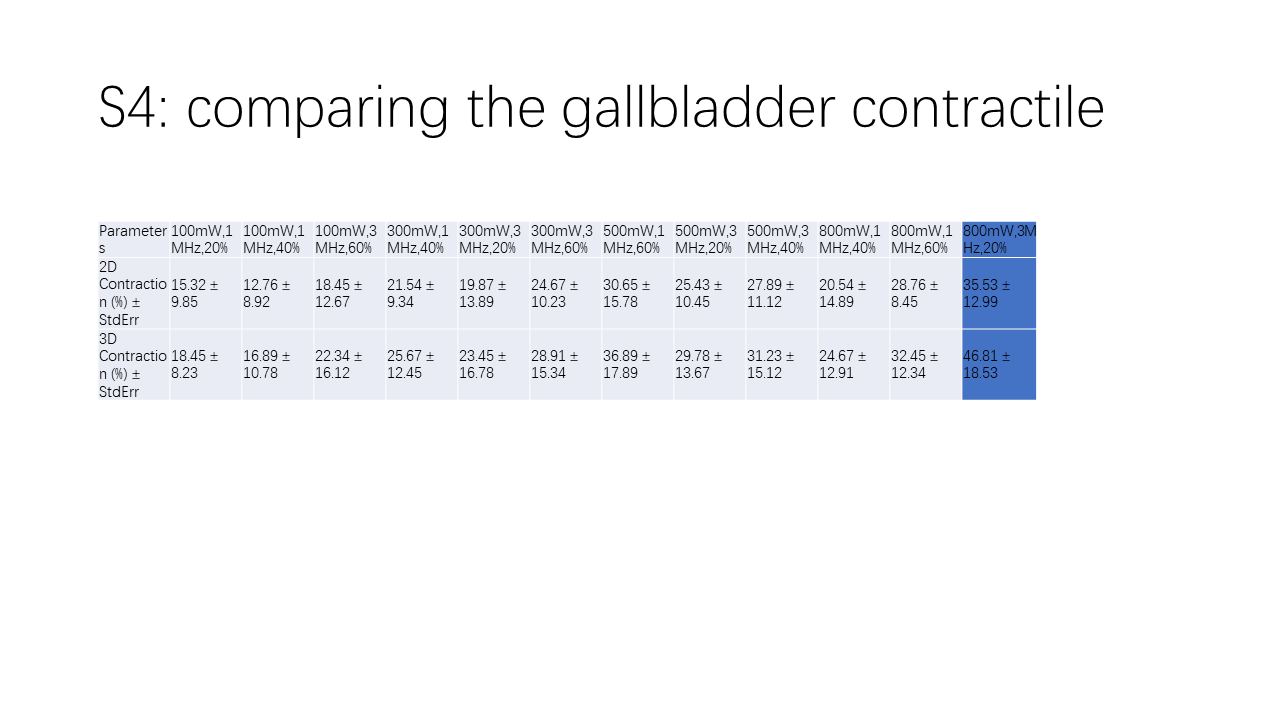

Supplement: Supplementary file 1 [file bioengineering-12-00716-s001.zip › S4.tif]
